# Enhancing mental wellbeing by changing mindsets? Results from two randomized controlled trials

**DOI:** 10.1186/s40359-023-01470-2

**Published:** 2024-02-15

**Authors:** Carina Schreiber, Marijke Schotanus-Dijkstra

**Affiliations:** 1 Department of Educational Sciences and Psychology, Center for Research on Education and School Development, Technical University Dortmund, Dortmund, Germany; 2 Faculty of Behavioural, Management and Social sciences; Department of Psychology, Health and Technology, University of Twente, P.O. Box 217, Enschede, 7500 AE The Netherlands

**Keywords:** Stress mindset, Mental wellbeing, Attributional beliefs, Philosophy of life, Flourishing mental health

## Abstract

Improving mental wellbeing is often targeted with behavioral interventions, while mindset interventions might be more appealing as they require less time and effort. In addition to recent experimental studies demonstrating that attributional beliefs can be changed to improve emotional wellbeing and performance, the current study examines whether a positive change in people’s beliefs about stress and life philosophy enhances emotional, social and psychological wellbeing using brief educational interventions. Two parallel double-blind randomized controlled trials were conducted. Study 1 (*N* = 106; 62.3% female, mean age 36.0) compared an educational video about the benefits of a stress-is-enhancing mindset versus an active control video. In Study 2 (*N* = 136; 57.4% female, mean age 35.7), educational texts about the benefits of a stress-is-enhancing mindset and holding a life-is-long-and-easy mindset were compared to an active control text. Results of multilevel growth curve modeling showed that a stress mindset could be significantly changed using an educational video or text, while the change in the philosophies of life mindset did not significantly differ between conditions. Furthermore, none of the manipulations were able to sustain the positive change in mindset which might explain why there was no significant increase in mental wellbeing compared to control. To have a lasting effect on people’s mental wellbeing, a change in mindset might need to be embodied in everyday life. Future research should investigate simple versus intensive interventions with longer follow-up time to examine whether and how a mindset can be sustainably changed to promote flourishing mental health in the general population.

**Supplementary Information:**

The online version contains supplementary material available at 10.1186/s40359-023-01470-2.

Improving mental wellbeing in the general population seems a major challenge in the 21st century. Performance pressure in education and occupation, the COVID-19 pandemic, and the rise of mental health problems are only some of the hindering factors to experience wellbeing (e.g., [1–3]). Although North and West European countries dominate the world ranking of the most happiest countries [4], much less of their citizens are able to flourish in life [5, 6]. Flourishing mental health can be viewed as the highest end of the mental wellbeing continuum, which consists of emotional wellbeing (i.e., happiness, life satisfaction, positive affect) [7], social wellbeing (e.g., social contribution, social acceptance) [8] and psychological wellbeing (e.g., personal growth, autonomy, self-acceptance) [9]. Hence, flourishing means more than feeling happy and satisfied with life [10]; people who flourish are also doing good for themselves and society [11, 12]. Cross-sectional studies have shown that flourishing is related to better work performance, fewer physical health complaints and lower health care costs [13, 14]. More importantly, longitudinal evidence revealed that flourishing reduced the risk of mortality and the onset and persistence of common mental disorders [15–18].

According to the Eudaimonic Activity Model, the most optimal path towards sustainable wellbeing and flourishing is to pursue growth-promoting goals and intentional behaviors with consistent effort [19–22]. An increase in happiness and positive affect is then thought to automatically follow. Nowadays, a wide variety of positive behavioral interventions have been developed which can effectively improve mental wellbeing in the general population [23–26]. Whether an individual can successfully change towards positive behavior depends, however, on aspects such as intrinsic motivation, time, effort, character traits like grit (i.e., a combination of being perseverant and having a passion for long-term goals), and social support [20, 27]. For example, a study among 340 undergraduate and postgraduate university students showed that those with higher levels of grit also scored higher on growth mindset and mental wellbeing [28]. Therefore, a more cost-effective approach might be to first endeavor altering people’s beliefs which in turn can affect their behavior. Indeed, positive beliefs could directly influence the way people behave, or they might align with (intended) positive behaviors in such a way that the actual behavior change can be achieved with less time and effort [29, 30]. As a consequence, disseminating how people can change their mindset might be more appealing for a wider population. While targeting people’s beliefs to enhance their wellbeing seems promising, to date, little is known about the impact and changeability of wellbeing related attributional beliefs.

Influential theoretical frameworks such as the Health Belief Model [31], the Theory of Planned Behavior [32, 33], the Cognitive Dissonance Theory [29] and more specifically, the Growth Mindset theory [34, 35] postulate that people’s beliefs influence their implicit and intentional behaviors. According to the latter theory, a mindset is “a mental frame or lens that selectively organizes and encodes information, thereby orienting an individual towards a unique way of understanding an experience and guiding one towards corresponding action and responses” ([36], p. 717 adapted from [35]). The theory and studies of Dweck focus on the belief that human traits and attributes are relatively stable (fixed mindset) versus the belief that human traits and attributes can develop and change incrementally through a person’s effort (growth mindset) [35]. The majority of studies on mindsets have focused on the malleability of intelligence and personality (e.g., [35, 37]), but scientific interest in the adaptability of other attributes is growing (e.g., [38, 39]). In particular, two types of mindsets might have the potential to influence people’s wellbeing, namely a stress mindset [36] and a philosophies of life mindset [40].

The stress mindset was introduced by Crum and her colleagues [36] and defined as “the evaluation of the nature of stress itself as enhancing or debilitating” (p. 718). In the following years, studies found that a stress-is-enhancing mindset is associated with increased life satisfaction and less symptoms of depression, anxiety and perceived stress compared to holding a stress-is-debilitating mindset (e.g., [36, 41–44]). Research also showed that a stress mindset could be changed successfully by means of a simple intervention [36, 41, 45]. By presenting a short video with evidence in favor of a stress-is-enhancing mindset, Crum and her colleagues [36] were able to change participants’ mindsets from a stress-is-debilitating mindset to a stress-is-enhancing mindset. More importantly, the change towards a more stress-is-enhancing mindset was accompanied by positive changes in individuals’ work performance and symptoms of anxiety and depression [36]. In another study, the stress mindset of university students was successfully changed to a more stress-is-enhancing mindset, but this change led only to improved positive and negative affect, perceived distress, proactive behavior and academic performance when perceived distress at baseline was high [41]. Yet, it is unknown whether a change to a stress-is-enhancing mindset could enhance people’s mental wellbeing rather than sole happiness or life-satisfaction.

Another mental frame that may be related to wellbeing is the life philosophy mindset. Based on a philosophical debate about Hobbes view that life is “nasty, brutish and short”, Norton and colleagues [40] explored whether people endorse the belief that life is short versus long and the belief that life is hard versus easy. Prior research regarding the belief that life is enduring has shown that if people were willing to donate some of their time to volunteer for a charity organization, they were also more likely to donate more money [46]. Similarly, a study showed that older employees with a higher subjective life expectancy had the intention to work longer [47]. In contrast, results from research about the perceived difficulty of life is less clear. For instance, despite the realization that effort often brings more pleasure in life [22, 48] and that a certain level of difficulty is needed for flow and personal growth [49], laypersons still desire an easy – yet meaningful – life [22]. Taken endurance and difficulty of life into account, Norton and colleagues [40] demonstrated that most participants from both North America and India held the belief that life is short and hard (45–61% across studies). Interestingly, the least popular view was that life is long and easy (6–15% across studies), while this mindset was associated with higher levels of happiness, life-satisfaction, volunteering, charitable donations and optimism about the future [40]. To our knowledge, the malleability of the philosophy of life mindset has not yet been investigated and potential effects of a shift to a life-is-long-and-easy mindset are unknown.

To summarize, the majority of people seem to hold a stress-is-debilitating mindset and a life-is-short-and-hard mindset. Based on the Eudaimonic Activity Model, Cognitive Dissonance Theory and the Growth Mindset Theory, it can be argued that a change in people’s beliefs might directly have an influence on their level of mental wellbeing. Consequently, this implies the potential efficacy of widespread, easy-to-administer, and cost-effective interventions targeted at reshaping prevailing belief systems to positively impact mental wellbeing. In fact, empirical evidence has shown that holding a more positive mindset is associated with several mental health related benefits, albeit mental wellbeing defined as having optimal levels of emotional, social and psychological wellbeing has not yet been investigated.

Therefore, the aim of the current paper is to examine whether a positive change in people’s beliefs about stress and life philosophy induced by simple educational interventions enhances mental wellbeing (i.e. emotional, social and psychological wellbeing). A series of two studies was conducted to test interventions with different delivery modes of information (i.e., video versus text), offering insights into the efficacy of different mediums in changing mindsets. In Study 1, an educational video in favor of a stress-is-enhancing mindset is compared to an active control video condition up to 4-weeks follow-up. It is hypothesized that people in the experimental condition significantly improve more towards a stress-is-enhancing mindset compared to people in the control condition. Furthermore, this shift towards a stress-is-enhancing mindset in the experimental condition is hypothesized to be accompanied by a significant increase in mental wellbeing, positive affect and locus of control, and a significant decrease in negative affect and perceived stress over time when compared to control. In Study 2, educational texts in favor of a stress-is-enhancing mindset or a life-is-long-and-easy mindset are compared to a control text condition up to 1-week follow-up. Hypotheses are that people in the experimental conditions improve significantly more towards a stress-is-enhancing mindset or a life-is-long-and-easy mindset respectively, and attain significantly higher levels of mental wellbeing over time compared to people in the control condition.

## Study 1

### Method

#### Design

A parallel double-blind randomized controlled trial was conducted in which participants were randomly allocated to either an experimental stress mindset manipulation video or an active control video condition (allocation ratio 1:1). Online surveys were assessed at four different time points to capture both adaptive and sustainable responses to the interventions: at baseline, at posttest directly after the manipulation, and at 1 and 4 weeks follow-up. All methods were carried out using the CONSORT and JARS guidelines [50, 51], and the experimental protocols were approved by the Ethics Committee of the University of Twente (no. 190,218 and no. 191,189).

#### Participants and procedure

Participants were recruited by six students of the University of Twente using convenience sampling. Participants had to be at least 18 years old and German-speaking. A power analysis in G*Power yielded a total required sample size of 124 participants to detect a small effect size (β = 0.80, α = 0.05, *d* = 0.30) for a 2*4 repeated measures analysis. Eligible participants (*N* = 184) received an email with the link to the informed consent procedure and the baseline assessment. 134 participants completed the baseline assessment and were randomly assigned to the stress mindset video (*n* = 67) or control video condition (*n* = 67) by an independent researcher using random numbers from randomizer.org. The final sample consisted of 106 participants because 28 participants were excluded for not watching the video. Drop-outs were significantly younger (*M*_*dropout*_ = 28.2, *SD* = 10.7; *M*_*completers*_ = 35.8, *SD* = 16.1; *t*(134) = 2.43, *p* = .017) and experienced more stress (*M*_*dropout*_ = 14.97, *SD* = 6.88; *M*_*completers*_ = 12.22, *SD* = 5.70; *t*(134) = -2.23, *p* = .028) compared to completers.

The final sample consisted of 54 participants in the stress mindset condition and 52 participants in the control condition. Mean age was 36 years (*SD* = 16.20) and slightly more than half of the participants were female (62.3%), higher educated (54.7%) and in paid employment (55.7%). No significant differences were found between the two conditions on demographics and baseline outcome measures except for perceived stress; participants in the stress mindset condition (*M* = 13.31, *SD* = 6.35) experienced significantly higher levels of perceived stress at baseline compared to those in the control condition (*M* = 11.08, *SD* = 5.70; *t*(104) = 2.05, *p* = .043).

At follow-up, the majority of the participants completed the surveys 1 week (94.3%) and 4 weeks (84.9%) after posttest. There were no significant differences between completers and drop-outs at any timepoint on any demographics or baseline measures (*p*s > 0.109).

#### Conditions

Both conditions received a 3-minute educational video aiming to deliver comprehensive and persuasive information to laypersons. While both videos were in German and similar in length and form, containing images and music, their content differed.

##### Stress mindset condition

The stress mindset condition received an educational video in favor of a stress-is-enhancing mindset. The video was originally developed by Crum, Akinola (45). For the purpose of the present study, German subtitles were added. In the video, scientific examples are given explaining how stress can enhance performance, health and mental wellbeing, and how this effect can be increased when believing in the positive aspects of stress. The video aimed to persuade participants to perceive stress as enhancing rather than debilitating.

##### Control condition

The control condition received an educational sham video about Kant’s ethical theory of the categorical imperative. The video was retrieved online from YouTube and states that according to the categorical imperative, people should act in such a way, that their behavior could become a general ethical rule. By giving the participants neutral yet scientific information about the categorical imperative, the video aimed to pose a neutral, non-manipulative equivalent to the experimental stress mindset condition.

#### Measures

##### Mental wellbeing

The 14-item Mental Health Continuum Short Form (MHC-SF) was used to measure emotional, social and psychological wellbeing [52]. Due to a mistake in designing the survey in Qualtrics, the MHC-SF was only administered at pretest, 1-week FU and 4-week FU, and not at posttest. Answers on items such as “During the past month, how often did you feel happy?” and “During the past month, how often did you feel that you liked most parts of your personality?” range from *never* (0) to *every day* (5). Higher total mean scores (0–5) indicate higher levels of mental wellbeing. The MHC-SF is frequently used due to its comprehensibility and good psychometric properties (α > 0.80) as shown among various samples of adolescents and adults in studies from all continents (e.g., [52–55]) as well as in the present study (α = 0.88).

##### Stress mindset

To check whether the experimental manipulation of participants’ stress mindset was successful, the Stress Mindset Measure (SMM) was used [36]. The 8-item questionnaire measures the extent to which an individual holds the mindset that the effects of stress are debilitating or enhancing. The SMM evaluates the participants’ general stress mindset (e.g., “The effects of stress are negative and should be avoided”) and signs and symptoms related to the debilitating and enhancing consequences of stress in the field of health and vitality, learning and growth, and performance and productivity (e.g., “Experiencing stress improves health and vitality”). The participants answered the items by rating the extent to which they agree or disagree with the given statements on a five-point Likert scale ranging from *strongly disagree* (0) to *strongly agree* (4). Total summed scores ranged from 8 to 40, with lower scores indicating a stress-is-debilitating mindset and higher scores a stress-is-enhancing mindset. The SMM proved to have good psychometric properties in the current study (α = 0.88).

##### Positive and negative affect

The 20-item Positive and Negative Affect Schedule (PANAS) was used to assess the extent to which an individual experienced positive affect (e.g., excited; proud) and negative affect (e.g., irritable; upset) in the last 24 h [56]. The items are rated on a five point scale, ranging from *not at all* (1) to *extremely* (5). Total summed scores ranged between 10 and 50 on each subscale, higher scores indicating higher levels of positive or negative affect. In the current study, acceptable reliability was found for negative affect (α = 0.75) and good reliability for positive affect (α = 0.85).

##### Locus of control

The 9-item Internal Locus of Control subscale of Levenson’s Multidimensional Locus of Control Scale [57] was used to assess individual’s degree of perceived internal control (e.g., “My life is determined by my own actions”). The items are rated on a seven-point Likert scale, ranging from *strongly agree* (0) to *strongly disagree* (6). Higher summed scores (0–54) indicate a higher tendency towards perceived internal locus of control. The questionnaire demonstrated acceptable reliability in the current study (α = 0.77).

##### Perceived stress

The 10-item Perceived Stress Scale (PSS-10) developed by Cohen [58] was used to assess the degree in which people consider events in their lives as stressful in the past month (e.g., “In the last month, how often have you felt nervous and stressed?”). Answers ranged from *never* (0) to *very often* (4) with higher summed scores (0–40) indicating higher levels of perceived stress. The questionnaire showed good psychometric properties in the present study (α = 0.84).

#### Statistical analyses

Changes in the outcome measures over time were examined using multilevel growth curve modeling in *R* (version 0.99.902, NLME package) to account for repeated measures nested within individuals [59]. It was hypothesized that changes would be nonlinear over time because participants watched a brief video only once. An unconditional growth curve model was specified with linear and quadratic changes over time, which was then compared with hypothesis-testing models. Time was centered on the second time point (posttest).

$$\begin{array}{l}\mathrm{Composite}\;\mathrm{model}:\;{\mathrm Y}_{\mathrm{ij}}\;=\;{\mathrm\gamma}_{00}+{\mathrm\gamma}_{10}{\mathrm{Time}}_{\mathrm{ij}}+{\mathrm\gamma}_{20}{\mathrm{Time}^2}_{\mathrm{ij}}\;({\mathrm\varepsilon}_{\mathrm{ij}}+{\mathrm\zeta}_{\mathrm{oi}}+{\mathrm\zeta}_{1\mathrm i}{\mathrm{Time}}_{\mathrm{ij}}+{\mathrm\zeta}_{2\mathrm i}{\mathrm{Time}^2}_{\mathrm{ij}})\\\mathrm{Level}\;1\;\mathrm{model}:\;{\mathrm Y}_{\mathrm{ij}}\;=\;{\mathrm\pi}_{0\mathrm i}+{\mathrm\pi}_{1\mathrm i}{\mathrm{Time}}_{\mathrm{ij}}+{\mathrm\pi}_{2\mathrm i}{\mathrm{Time}^2}_{\mathrm{ij}}+{\mathrm\varepsilon}_{\mathrm{ij}}\\\mathrm{Level}\;2\;\mathrm{models}:\;{\mathrm\pi}_{0\mathrm i}\;=\;{\mathrm\gamma}_{00}+{\mathrm\zeta}_{0\mathrm i},\;{\mathrm\pi}_{1\mathrm i}\;=\;{\mathrm\gamma}_{10}+{\mathrm\zeta}_{1\mathrm i},\;\mathrm{and}\;{\mathrm\pi}_{2\mathrm i}\;=\;{\mathrm\gamma}_{20}+{\mathrm\zeta}_{2\mathrm i}\end{array}$$

### Results and discussion

**Fig. 1:**
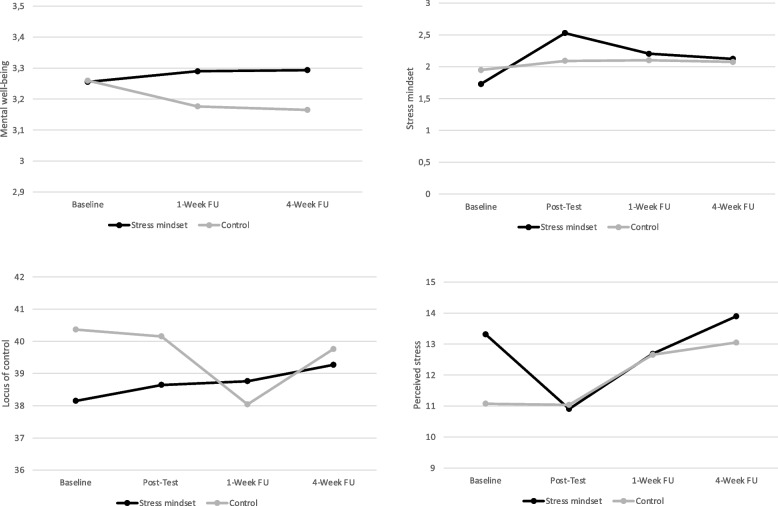
Average changes in mental wellbeing, stress mindset, locus of control and perceived stress by video condition (Study 1)

Results demonstrated that the stress-is-enhancing mindset increased significantly more over time in the stress mindset condition compared to control, *γ*_11_ = 0.25, S.E. = 0.05, *t*(292) = 4.57, *p* < .001; *γ*_21_ = -0.19, S.E. = 0.04, *t*(292) = -4.76, *p* < .001 (see Table 1). This effect of the educational video was mainly visible directly at posttest (*d* = 0.64, see Fig. 1). However, no linear or quadratic changes were found for mental wellbeing, positive affect and negative affect (*γ*s < 0.38, *p*s > 0.362). By contrast, linear changes over time were found for locus of control, *γ*_11_ = 1.12, S.E. = 0.49, *t*(292) = 2.28, *p* = .023 and perceived stress, *γ*_11_ = -1.17, S.E. = 0.48, *t*(289) = -2.42, *p* = .016, in favor of the stress mindset condition. Within this condition, locus of control gradually increased over time, while perceived stress reduced most strongly between pretest and posttest (see Fig. 1). Notably, between-group effect sizes per time point were not significant for locus of control and perceived stress (see Table 1). Overall, the findings indicate that the stress-mindset video had a positive – but small and temporary – effect on stress or mindset related outcomes, but no effect on mental wellbeing and general affect.

The findings of Study 1 underscore earlier findings about the changeability of stress mindsets [36, 41, 45]. As seen before [45], a sharp increase towards a stress-is-enhancing mindset was visible directly after watching an educational video in favor of this mindset compared to control. This sharp increase could perhaps have been more stronger when those participants with higher levels of stress at baseline were also included in the whole study, but for some unknown reasons they did not watch the manipulation videos and had to be excluded from the analyses. Although the overall stress-mindset trajectories over time differed between conditions in favor of the stress mindset condition, they only differed significantly at posttest and not at 1-week and 4-week follow-up. Hence, it seems that a mindset can quite easily be changed [36, 41, 45, 60, 61], but that a simple video manipulation might not be sufficient to sustainably maintain this change over a longer period of time.

In addition, Study 1 showed that the change towards a stress-is-enhancing mindset did not significantly led to more improvements in mental wellbeing compared to control. A possible explanation for this unexpected finding is that a potential steep increase in mental wellbeing immediately after the manipulation was missing from the growth curve analysis due to a constructional error at posttest. However, positive and negative affect, an important dimension of emotional wellbeing, did also not change significantly over time compared to control. Accordingly, a change in one’s mindset might merely fuel changes in proximal variables such as perceived stress and work performance when changing one’s stress mindset [36], or motivation and academic achievement when changing towards a growth mindset [62]. Because mental wellbeing consists of emotional, social and psychological wellbeing, it might be too distal from the belief that stress can be beneficial for one’s (physical) health and performance.

Therefore, in Study 2, we added a philosophy of life mindset condition, which seems more proximately related to mental wellbeing. We also used actively reading educational texts instead of passively watching videos because studies from other mindset types have shown that using educational or persuasive texts were successful in changing people’s mindset [38, 39]. Such texts may be exemplary to slightly increase participants’ effort in order to change their mindsets without approaching the effort that is usually needed to modify behavior. Moreover, educational texts have not yet been used in the field of stress mindset.

**Table 1:** Raw means (SDs) for all outcomes on each time point by condition and between-group Cohen’s d effect sizes (Study 1)

	Stress Mindset Video (*n* = 54)		Control Video (*n* = 52)		Between group effect size
	*n*	*M (SD)*	*n*	*M (SD)*	*d [95% CI]*
Mental Wellbeing^a^					
Pretest	54	3.26 (0.78)	52	3.26 (0.67)	
1-week FU	51	3.29 (0.89)	49	3.18 (0.59)	0.16 [-0.24–0.55]
4-week FU	48	3.29 (0.81)	42	3.17 (0.68)	0.18 [-0.24–0.59]
Stress Mindset					
Pretest	54	1.73 (0.79)	52	1.95 (0.59)	
Posttest	54	2.52 (0.74)	52	2.09 (0.64)	**0.64 [0.25–1.03]**
1-week FU	51	2.20 (0.79)	49	2.10 (0.60)	0.15 [-0.24–0.54]
4-week FU	48	2.12 (0.71)	42	2.07 (0.65)	0.07 [-0.34–0.49]
Positive Affect					
Pretest	54	34.54 (6.75)	52	33.37 (6.41)	
Posttest	54	34.50 (6.69)	52	32.12 (5.93)	0.37 [-0.02–0.75]
1-week FU	51	35.04 (7.74)	49	33.06 (5.49)	0.30 [-0.09–0.70]
4-week FU	48	34.65 (7.25)	42	33.17 (6.71)	0.23 [-0.19–0.64]
Negative Affect					
Pretest	54	16.59 (4.79)	52	16.25 (4.50)	
Posttest	54	15.48 (5.72)	52	15.17 (4.73)	0.06 [-0.32–0.44]
1-week FU	51	17.16 (6.23)	49	16.39 (4.86)	0.15 [-0.24–0.54]
4-week FU	48	17.06 (6.12)	42	16.07 (4.87)	0.19 [-0.22–0.61]
Locus of Control					
Pretest	54	38.15 (7.48)	52	40.37 (5.89)	
Posttest	54	38.65 (8.51)	52	40.15 (5.53)	-0.21 [-0.59–0.17]
1-week FU	51	38.76 (8.74)	49	38.04 (7.12)	0.10 [-0.29–0.49]
4-week FU	48	39.27 (7.74)	42	39.76 (6.06)	-0.07 [-0.48–0.35]
Perceived Stress					
Pretest	54	13.31 (6.35)	52	11.08 (4.74)	
Posttest	54	10.91 (7.15)	51	11.04 (5.98)	-0.02 [-0.40–0.36]
1-week FU	51	12.69 (7.13)	49	12.65 (5.64)	0.01 [-0.39–0.40]
4-week FU	48	13.90 (7.03)	40	13.05 (4.83)	0.14 [-0.28–0.55]

^a^Due to a mistake in designing the survey in Qualtrics, the MHC-SF measuring mental wellbeing was not included at posttest

## Study 2

### Method

#### Design

In the second study, a randomized controlled trial was conducted in which participants were randomly allocated to reading an educational text about either a stress-is-enhancing mindset, a life-is-long-and-easy mindset or about personality traits (active control condition) with an allocation ratio of 1:1:1. Online surveys were assessed at three different time points: at baseline, at posttest directly after reading the educational text and at 1-week follow-up. A 4-week follow-up was omitted due to disappointing recruitment for the available time and more attrition than anticipated.

#### Participants and procedure

Participants were recruited by eight students of the University of Twente using convenience sampling. The preconditions and procedure were similar to Study 1. The initial power analysis in G*Power yielded a total required sample size of 222 participants to detect a small effect size (β = 0.80, α = 0.05, *d* = 0.25) for a 3*4 repeated measures analysis. Of the 204 eligible participants, 155 participants completed the baseline survey and were randomly assigned to the stress mindset condition (*n* = 52), the life philosophy mindset condition (*n* = 51) or the active control condition (*n* = 52). The final sample consisted of 136 participants because 19 participants were excluded for not reading the text. Drop-outs were significantly younger (*M*_*dropout*_ = 25.5, *SD* = 7.3; *M*_*completers*_ = 35.7, *SD* = 16.5; *t* (152) = 4.57, *p* = < 0.001) compared to completers, but they did not differ on any other demographics or outcome measures (*p*s > 0.112).

The final sample consisted of 45 participants in the stress mindset condition, 47 participants in the philosophy of life mindset condition, and 44 participants in the control condition. The mean age of the final sample was 35.7 (*SD* = 16.5) and slightly more than half of the participants were female (57.4%), intermediately educated (61.0%) and in paid employment (58.8%). No significant differences were found between the three conditions on demographics and baseline outcome measures (*p*s > 0.337). The majority of the participants completed the 1-week follow-up (90.4%) and no significant differences were found between drop-outs and completers although those in paid employment were marginally less likely to drop-out at follow-up, *χ*^*2*^(2) = 5.53, *p* = .063.

#### Conditions

All three conditions received a text to read, aiming at delivering comprehensible and convincing information to laypersons. All three texts were in German and similar in length, but their content differed (see Supplemental Material for full details).

##### Stress mindset condition

Participants were instructed to read an educational text in favor of a stress-is-enhancing mindset. By referring to scientific evidence about the beneficial effects of stress on energy levels, workplace performance, life satisfaction and psychological symptoms the text aimed to persuade participants to believe in the positive nature of stress and to perceive it as enhancing rather than debilitating. This educational text was based on results from a study by Crum and colleagues [36].

##### Philosophy of life mindset condition

Participants in this condition were instructed to read a text about the benefits of holding a life-is-long-and-easy mindset in contrast to a life-is-short-and-hard mindset on wellbeing, relationship satisfaction and happiness. This educational text was based on results from a study by Norton and colleagues [40], aimed to change participants mindset, but regarding a positive life philosophy rather than positive beliefs about stress.

##### Control condition

Participants in the active control condition received a neutral, educational text about the Big Five. Participants were informed that the Big Five are not only game animals in Africa but also the core traits used to describe people’s personality. By providing scientific information about the Big Five personality dimensions, it was expected that this text was of interest to participants but unlikely to change participants mindset about stress or life philosophies.

#### Measures

Similar to Study 1, mental wellbeing was measured with the MHC-SF (α = 0.90) and stress mindset with the SMM (α = 0.87) at all time points.

In addition, the philosophies of life mindset was measured with two items derived from Norton and his colleagues [40] at all time points. First, “Is life short, or long?” and second, “Is life easy, or hard?”. This resulted in four different life philosophies: (1) life is long and easy, (2) life is short and easy, (3) life is long and hard, and (4) life is short and hard [40]. In the present study, a change score was calculated by assigning participants to one of the following two groups. First, an optimistic change group was created including those who stayed in the life-is-long-and-easy philosophy or changed to this philosophy at posttest. Second, a pessimistic change group consists of those who stayed in one of the other three philosophies or changed to one of those three philosophies (i.e., life-is-short-and-easy, long-and-hard, or short-and-hard).

#### Statistical analyses

To assess changes over time, the same statistical analyses were conducted as described in Study 1. In addition, chi-square tests and planned contrasts were used to examine the changes in life philosophies over time and in relation to mental wellbeing between conditions.

### Results and discussion

**Fig. 2:**
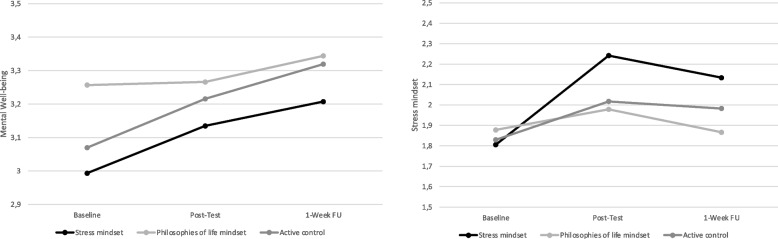
Average changes in mental wellbeing and stress mindset by text condition (Study 2)

Results of multilevel growth curve modeling showed that the stress-is-enhancing-mindset significantly increased in the stress mindset condition, but mainly compared to the philosophies of life condition, *γ*_11_ = 0.14, S.E. = 0.06, *t*(253) = 2.50, *p* = .013; *γ*_21_ = -0.19, S.E. = 0.08, *t*(253) = -2.35 *p* = .019, and only marginally compared to control *γ*_11_ = 0.09, S.E. = 0.06, *t*(253) = 1.48, *p* = .140; *γ*_21_ = -0.16, S.E. = 0.08, *t*(253) = -1.93, *p* = .055 (see Table 2). Participants stress-is-enhancing mindset increased most strongly directly after reading the text (*d* = 0.59; see Fig. 2) and gradually decreased up to 1-week follow-up (*d* = 0.15).

A Pearson chi-square test revealed that most participants endorsed the life-is-short-and-hard philosophy (37.5%), while the popularity of the life-is-long-and-easy philosophy (22.1%) was comparable to having a short-and-easy (21.3%) and long-and-hard philosophy (19.1%), c^2^ (1) = 4.02, *p* = .045. At posttest, mainly those in the philosophies of life mindset condition changed their life philosophy to long-and-easy from 19.1% at pretest to 36.2% at posttest and 38.6% at follow-up (see Table 2). However, optimistic changes did not significantly differ between conditions at posttest, c^2^ (2) = 2.80, *p* = .247, or follow-up, c^2^ (2) = 2.00, *p* = .368, probably a result of an optimistic change in the control condition as well.

In line with expectations, mental wellbeing was significantly higher among those endorsing a life-is-long-and-easy philosophy at pretest (*M* = 3.47, *SD* = 0.57) compared to those endorsing a life-is-short-and-hard philosophy (*M* = 2.79, *SD* = 0.97), *t*(79) = 3.99, *p* < .001 (see Table 3). Similar results were found at posttest, *t*(132) = 2.73, *p* = .009, and at 1-week follow-up, *t*(119) = 3.27, *p* = .001. However, mental wellbeing was not significantly higher among participants who optimistically changed their philosophies of life mindset directly after reading the educational text, *t*(134) = 1.38, *p* = .171. By contrast, one week after reading the text, those who optimistically changed towards a life-is-long-and-easy philosophy had higher levels of mental wellbeing (*M* = 3.59, *SD* = 0.75) compared to those with a pessimistic change (*M* = 3.15, *SD* = 0.96), *t*(121) = 2.50, *p* = .014. Nevertheless, when comparing the three conditions in multilevel analyses, no linear or quadratic changes for mental wellbeing were found in favor of any of the conditions (*γ*s < 0.12, *p*s > 0.103).

Study 2 adds to prior literature [36, 41, 45] that a stress mindset can also be changed by reading an educational text. Effect sizes of Study 1 and 2 are comparable, but direct comparison between a manipulative video and text is needed to examine whether and when a certain delivery mode is preferred in order to change one’s mindset successfully. Expanding on prior results, the current study also showed that participants who held a life-is-long-and-easy mindset possessed higher levels of mental wellbeing rather than sole happiness and life satisfaction [40].

However, the manipulation did not led to a significant change towards a life-is-long-and-easy mindset in comparison with control. Additionally, mental wellbeing did not significantly increase more over time in favor of any of the experimental conditions which is in line with Study 1. Brief educational videos and texts seem, therefore, not sufficient to change one’s mindset in a way that it could enhance people’s mental wellbeing. A possible explanation might be the relatively low number of participants per condition which might have resulted in reduced statistical power. More likely is that the text about personality in the control condition – perhaps in combination with completing surveys about mindsets and mental wellbeing – might unintentionally have been effective in the control condition as indicated by more favorable life philosophies and increased mental wellbeing over time within this group. For example, the recurring assessments about life philosophies might have triggered respondents in the control condition to reflect on whether life is long, short, easy or hard. Unconscious beliefs or deliberate reflection might have evoked a shift in mindset, and might have limited sufficient comparison between the three conditions.

Another explanation is that the baseline beliefs about the philosophy of life were already more favorable in the current study compared to baseline beliefs in the study of Norton and colleagues [40]. More specifically, less participants in the current study believed that life is short and hard (38% vs. 45–61%) and more participants thought of it as long and easy (22% vs. 6–15%). Thus, fewer participants in the present study had room to optimistically change their philosophies of life mindset – and assumed corresponding levels of mental wellbeing – compared to prior research. Taken together, Study 2 implies that a stress mindset can be changed with minimal effort, but that the use of simple texts are insufficient to change people’s mental wellbeing.

**Table 2:** Raw data for outcomes on each assessment by condition (Study 2)

	*n*	Stress Mindset	*n*	Philosophies of Life Mindset	*n*	Control Condition
Mental wellbeing, M (SD)						
Pretest	45	2.99 (0.94)	47	3.26 (0.81)	44	3.07 (0.88)
Posttest	45	3.13 (0.98)	47	3.26 (0.93)	44	3.22 (0.97)
1-week FU	43	3.21 (0.87)	44	3.34 (0.93)	36	3.32 (0.98)
Stress Mindset, M (SD)						
Pretest	45	1.81 (0.74)	47	1.88 (0.82)	44	1.83 (0.65)
Posttest	45	2.24 (0.75)	47	1.98 (0.74)	44	2.02 (0.63)
1-week FU	43	2.13 (0.86)	44	1.87 (0.76)	36	1.98 (0.81)
Long and Easy Philosophy, n (%)						
Pretest	45	13 (28.9)	47	9 (19.1)	44	8 (18.2)
Posttest	45	14 (31.1)	47	17 (36.2)	44	9 (20.5)
1-week FU	43	12 (27.9)	44	17 (38.6)	36	9 (25.0)
Short and Easy Philosophy, n (%)						
Pretest	45	10 (22.2)	47	11 (23.4)	44	8 (18.2)
Posttest	45	9 (20.0)	47	11 (23.4)	44	12 (27.3)
1-week FU	43	10 (23.3)	44	10 (22.7)	36	11 (30.6)
Long and Hard Philosophy, n (%)						
Pretest	45	9 (20.0)	47	8 (17.0)	44	9 (20.5)
Posttest	45	8 (17.8)	47	11 (23.4)	44	8 (18.2)
1-week FU	43	8 (18.6)	44	6 (13.6)	36	4 (11.1)
Short and Hard Philosophy, n (%)						
Pretest	45	13 (28.9)	47	19 (40.4)	44	19 (43.2)
Posttest	45	14 (31.1)	47	8 (17.0)	44	15 (34.1)
1-week FU	43	13 (30.2)	44	11 (25.0)	36	12 (33.3)

**Table 3:** Means and SD’s for the level of mental wellbeing by each philosophy of life mindset throughout Study 2

	Philosophies of life							
	*n*	Long-and-easy *M (SD)*	*n*	Short-and-easy *M (SD)*	*n*	Long-and-hard *M (SD)*	*n*	Short-and-hard *M (SD)*
Mental wellbeing								
Pretest	30	3.47 (0.57)	29	3.44 (0.72)	26	2.95 (0.89)*	**51**	2.79 (0.97)***
Posttest	**40**	3.38 (0.84)	32	3.67 (0.73)	27	2.93 (1.06)*	37	2.82 (0.97)**
1-week FU	**38**	3.59 (0.75)	31	3.50 (0.82)	18	3.02 (0.99)*	36	2.92 (0.99)**

*p < .05 compared to the long-and-easy mindset

**p < .01 compared to the long-and-easy mindset

***p < .001 compared to the long-and-easy mindset

## General discussion

The current study adds to prior research by examining the efficacy of changing people’s mindset beyond emotional wellbeing and performance, by including not only a stress mindset condition but also determining the possibilities of changing one’s life philosophy mindset, and by including different modes of delivery, namely educational videos and texts. The results confirm that most people endorse a stress-is-debilitating mindset and a life-is-short-and-hard mindset. The study also implies that a stress mindset can be changed quite easily, with an educational video or text, and that holding a life-is-long-and-easy mindset is associated with enhanced mental wellbeing. However, we did neither find evidence for a sustainable change in mindsets nor an improvement in people’s mental wellbeing over time compared to control. In fact, these results seem intertwined as participants’ shift towards a stress-is-enhancing mindset or a life-is-long-and-easy mindset receded within the first week after the manipulation, a time span which might have been too short for the shift in mindsets to have an effect on people’s mental wellbeing [25, 26]. Since flourishing mental wellbeing is a conjunction of expedient feelings, thoughts and behaviors, the impact of a newly acquired mindset on mental wellbeing related behaviors might become visible only after people have embodied this mindset in everyday life. This is supported by the finding that those who possessed more positive mindsets at baseline showed higher levels of mental wellbeing when compared to those who did not.

A possible explanation for not finding a sustainable change in mindsets is that the manipulation tasks may have been too easy for participants, resulting in minor or temporary feelings of cognitive dissonance. Theoretically, a change in peoples mindset could lead to inconsistencies when the newly acquired belief encounter old beliefs or behaviors [29]. To re-enforce harmony, people are likely to change these dissonant beliefs and behaviors by either adapting towards the newly acquired mindset (e.g., “Life is long and easy, so why shouldn’t I just take the time to read the newspaper this morning”), or to discard the new mindset and revert to their initial beliefs and behaviors (e.g., “I was right, life is indeed short and hard, so I really start with my bucket-list now”). The effort-justification paradigm in particular states that people are more likely to adapt their old beliefs into a new mindset when this mindset is obtained by engaging in more unpleasant and effortful activities [30].

In line with this, a previous study maintained a beneficial shift in participants stress mindset up to two weeks after the manipulation [63] by using a more demanding experiment that consisted of watching a series of videos, two mental imagery exercises and a writing task about the positive consequenses of stress. In contrast, an experimental study determining the malleability of people’s healthy eating mindset revealed that a more intensive workshop in which participants experienced the sensory attributes of healthy nutrition (e.g. indulgent, pleasurable, social) was only successful directly after the workshop and not at follow-up [39]. Thus, the current knowledge yield contradicting results regarding the dose-response relationship of interventions targeting changes in mindsets. Indeed, there is also some promising evidence that simple educational text interventions can be effective in sustainably changing mindsets [38, 39]. Hence, these inconsistencies in the dose-response relationship limit a firm conclusion about people’s effort needed when aiming for a lasting change in mindsets.

Another possible explanation for not finding a sustainable change in mindsets and assumed corresponding flourishing mental health could be the unilateral exposure to the mindsets under study. Life can be hard and stressful at times, and certain situations legitimate a debilitating view on stress or life in general [64]. Participants might have disregarded the merely positive side of stress or life manipulations and may have considered it as unrealistic based on personal negative experiences. More balanced educational interventions displaying more naturalistic beliefs about stress and life philosophy might promote a change in people’s mindsets more deliberately and sustainably [41, 65]. For instance, a prior study demonstrated that a sole focus on either positive or negative outcomes of stress reduced the use of effective coping strategies in comparison with those who learned about a balanced view on stress [65]. Such balanced views could also facilitate cognitive dissonance in participants because they could become more consciously aware of the gap between their own black-and-white beliefs versus more beneficial and nuanced beliefs.

### Limitations

The two experiments should be interpreted in light of the following limitations. Firstly, the current sample sizes were sufficient but minimal, especially after excluding participants who did not watch the manipulative video or read the manipulative text. Secondly, participants were all recruited via the network of several students from the same academic study program, which limits the generalizability of the findings. Lastly, the manipulative texts have not been tested in a pilot study, for example by using the think-aloud method [66, 67]. As a result, it is unclear how participants understood, interpreted and processed the information.

### Directions for future research

More research is needed to establish effective ways to sustainably change people’s beliefs which could facilitate more eudaimonic behaviors and subsequent mental wellbeing. A first step is to encourage researchers to use longer follow-up periods within mindset research to examine how long a change in mindset can last with associated benefits for people’s mental health. To date, the majority of studies are cross-sectional or lack follow-up measurements after an experiment [36, 42, 45, 68, 69]. A second avenue for future mindset studies is to investigate the ideal parameters of manipulations to facilitate sustainable changes in mindsets. Thus, comparing more different manipulation tasks such as varying between more or less effort and pleasurable activities. Examples are exposure to a severe stress activity or solving impossible puzzles before a reading and writing task about the positive consequences of stress or life philosophy and a repetition of brief educational videos, texts or workshops with more balanced knowledge on stress or the philosophy of life. A third direction for future research is to conduct ecological momentary assessment studies to examine how people perceive stressful situations before, during and after a manipulative video, text or intervention program in the real-life context. Finally, examples from nudging and in particularly priming research in other fields such as education, might inspire research within the field of psychological mindsets as well [70].

## Conclusion

In conclusion, motivating people to change their beliefs towards a stress-is-enhancing mindset and in particular a life-is-long-and-easy mindset could be a fruitful direction to enhance mental wellbeing in the general population. In particular can the stress mindset be changed relatively easy using simple video and text manipulations albeit more effort is needed to change mindsets sustainably to ensure a change towards flourishing mental health as well. Future mindset scholars should prioritize the use of longitudinal assessments along a wide variety of simple and advanced mindset interventions. Researchers in this field should also consider mental wellbeing as primary outcome rather than sole aspects of emotional wellbeing.

### Supplementary Information

**Additional file 1.**

## Data Availability

The data used for this study is available via 10.17026/SS/DFEXWO. Questions about these open source data files can be addressed to the corresponding author.
