# Supplementary material for: Enhancing mental wellbeing by changing mindsets? Results from two randomized controlled trials
Source: BMC Psychol. 2024 Feb 15;12:77. doi: 10.1186/s40359-023-01470-2 (PMC10870491; doi:10.1186/s40359-023-01470-2)
Supplement: Supplementary file 1 — Additional file 1. [file 40359_2023_1470_MOESM1_ESM.docx]

**Manipulation texts of Study 2**

The following texts were used in the three conditions of Study 2, namely the stress mindset condition, life philosophies condition and active control condition. These texts were used in German but translated in English for clarity. German texts are available upon request.

1. **Stress Mindset Condition**

Did you know that stress is beneficial for your health and personal growth? Although stress is being portrayed in a negative way in the media and by the people around us, there is also a positive side of experiencing stress. For example, people who believe that stress is positive have higher energy levels, show better workplace performance, are more satisfied with their life in general and have fewer symptoms of depression and anxiety. How do you interpret a stressful situation? Do you find stress negative or positive?

Recent scientific studies have shown that experiencing stress puts the body and the brain in an optimal condition to function in order to fulfill the demands and tasks asked for. Therefore, the attention is focused on the demands and this will boost memory and performance. Stress is an essential ingredient of being able to fulfill everyday tasks as well as more difficult challenges. Thus, individuals who perceive stress as a necessary and positive aspect of life are more likely to succeed and feel happy.

Taken together, if you believe that stress is positive, this can have a great beneficial impact on your personal growth, performance and your health.

1. **Life Philosophies Condition**

Did you know that your perspective of life influences your health and behavior? How do you perceive life? Do you think that life is short or long? And do you think that life is easy or hard?

Recent scientific studies have shown that most people believe that life is short and hard. However, those people who believe that life is long and easy have better health outcomes. In general, they do not only possess a higher level of well-being, they also feel more happy, donate more money to charity, do more often volunteer work and are more satisfied with their relationships compared to the individuals who are holding another view of life (namely, that life is long and hard, short and hard or short and easy). Also, individuals who believe that life is long and easy think that they will experience less worse and more good things to happen in the future compared to individuals who possess the short and hard view of life.

Taken together, if you believe that life is long and easy, you are more likely to feel better and do better, for example by improving the well-being of others.

1. **Active Control Condition**

Did you know that ‘The Big Five’ are not only animals but also indicate your personality? While the big five animals in Africa refer to the five animals most difficult to hunt on foot - the lion, leopard, rhinoceros, elephant and cape buffalo - psychologists use the term to describe the five core traits of your personality:

1. Openness to experience: curious, broad range of interests, try new things.
2. Conscientiousness: thoughtfulness and planning, organized, attention to detail.
3. Extraversion: sociable, talkative, assertive, outgoing and energized.
4. Agreeableness: trust, kindness, cooperative, care about other people.
5. Neuroticism: emotional unstable, mood swings, gets upset easily.

Recent scientific studies have shown that both biological and environmental influences play a role in shaping our personalities. Studies also suggest that these big five personality traits tend to be relatively stable over the course of adulthood. It is important to note that each of the five personality factors represents a range between two extremes. For example, extreme extraversion versus extreme introversion, and neuroticism (emotional instability) versus emotional stability. In the real world, most people lie somewhere in between the two polar ends of each dimension.

Taken together, your personality can be categorized into five main personality traits which are relatively stable.
